# Supplementary material for: Unbiased Strain-Typing of Arbovirus Directly from Mosquitoes Using Nanopore Sequencing: A Field-forward Biosurveillance Protocol
Source: Sci Rep. 2018 Apr 3;8:5417. doi: 10.1038/s41598-018-23641-7 (PMC5883038; doi:10.1038/s41598-018-23641-7)
Supplement: Supplementary file 1 — Supplementary Materials [file 41598_2018_23641_MOESM1_ESM.docx]

# **Unbiased Strain-Typing of Arbovirus Directly from Mosquitoes Using Nanopore Sequencing: A Field-forward Biosurveillance Protocol**

Joseph A. Russell^1^, Brittany Campos^2^, Jennifer Stone^2^, Erik M. Blosser^3^, Nathan Burkett-Cadena^3^, Jonathan L. Jacobs^1*^

1. MRIGlobal, 65 West Watkins Mill Road, Gaithersburg, MD, USA 20878

2. MRIGlobal, 1470 Treeland Blvd. SE, Palm Bay, FL, USA 32909

3. University of Florida - Florida Medical Entomology Laboratory, 200 9^th^ St. SE, Vero Beach, FL, USA 32962

* jjacobs@mriglobal.org

# Supplementary Material

## Bioinformatics Software Parameters

### ------LAST------

$ lastdb -Q 0 VEEV_reference_genomes VEEV_reference_genomes.fasta

$ lastal -s 2 -T 0 -a 1 -Q 1 -f BlastTab VEEV_reference_genomes data.fastq > data_alns.maf

$ grep "^[^#;]" data_alns.maf | awk -F '\t' '{print $1}' | sort | uniq -c | sort -nr | wc -l

### ------BWA-MEM------

$ bwa index VEEV_reference_genomes.fasta

$ bwa mem -x ont2d VEEV_reference_genomes.fasta data.fastq > data.sam

$ samtools view -bS data.sam > data.bam

$ samtools sort data.bam -o data_sorted.bam

$ samtools view -F 260 data_sorted.bam | cut -f 3 | sort | uniq -c | awk '{printf("%s\t%s\n", $2, $1)}' > counts.txt

### ------CENTRIFUGE------

$ set -xeu

$ /src/centrifuge/centrifuge –x /src/centrifuge/indices/phv -U data.fastq -S data.out -p 16 --met-stderr

### ------KRAKEN------

$ kraken --preload \

--db /home/src/kraken/full \

--fastq-input \

--threads 16 \

--classified-out ./$DATA-class.fa \

--unclassified-out ./$DATA-unclass.fa \

--output ./$DATA-krakenout.txt \

data.fastq

$ kraken-report --db /home/src/kraken/full ./$DATA-krakenout.txt > ./$DATA-kreport.txt

$ kraken-mpa-report --db /home/src/kraken/full ./$DATA-krakenout.txt > ./$DATA-mpkraken.txt;

### ------KAIJU------

$ set -xeu

$ kaiju -t /home/src/kaiju/bin/kaijudb/nodes.dmp -f /home/src/kaiju/bin/kaijudb/kaiju_db.fmi -i data.fastq -o ./data.kaiju.out -v -z 16 -a greedy -e 10 -s 35

$ addTaxonNames -t /home/src/kaiju/bin/kaijudb/nodes.dmp -n /home/src/ kaiju/bin/kaijudb/names.dmp -i ./data.kaiju.out -o ./data.kaiju-names.out

$ kaijuReport -t /home/src/kaiju/bin/kaijudb/nodes.dmp -n /home/src/kaiju/bin/kaijudb/names.dmp -i ./data.kaiju.out -r species -o ./data.kaiju-names.out.summary

## SUPPLEMENTAL FIGURES

**Supplementary Figure S1**: Two examples of chimeric REPLI-g generated nanopore reads from virus-positive mosquito pool sample 4.1. Illumina MiSeq reads that mapped to any strain in the custom VEEV database were isolated and re-mapped to REPLI-g generated nanopore reads that also aligned to VEEV references in CLC-Bio Genomics Workbench v. 10.0.1. One would expect generally uniform distribution of re-mapped MiSeq reads across VEEV-associated nanopore reads. However, MiSeq reads are observed to align with specific regions of nanopore reads, and are absent from other regions, indicating chimerism in REPLI-g generated nanopore reads. (**A)** The purple-highlighted region of Channel_284_read_6073 was BLASTed against the nt database and the highest associated hits were for the mosquito *Culex quinquefasciatus* and *Drosophila* spp. (**B**) The purple highlighted region of Channel_102_read_4542 returned *Culex quinquefasciatus* and *Aedes aegypti* as top hits in its BLAST result.


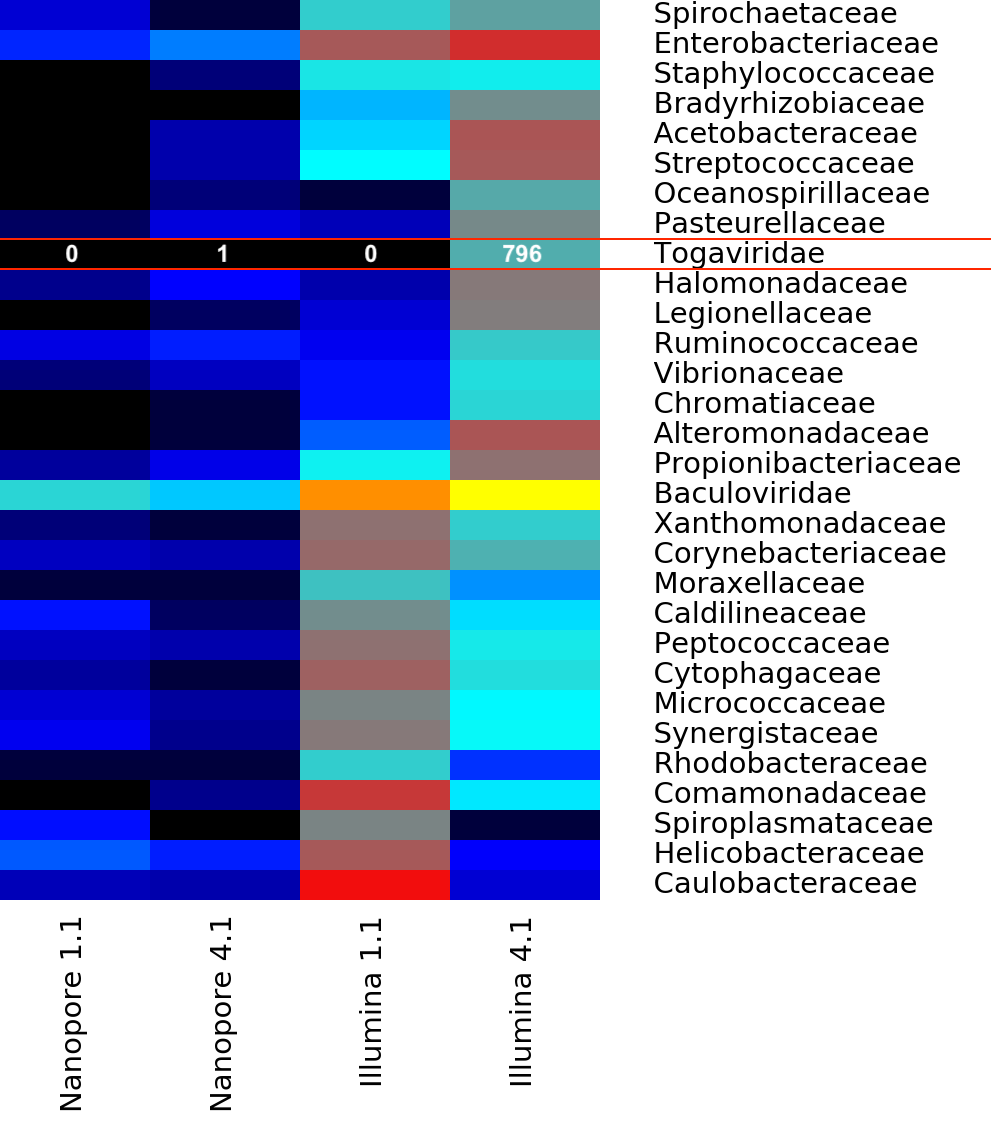


**Supplementary Figure S2:** Bacterial and viral community profiles of the top 30 genera identified from each sample, via both Illumina and Nanopore sequencing, as classified by *kraken*. The number of reads classified by *kraken* are boxed in red and shown for *Togaviridae*, the viral family that includes Everglades virus. The complete results at the family level are shown in Supplementary Table 2 below.


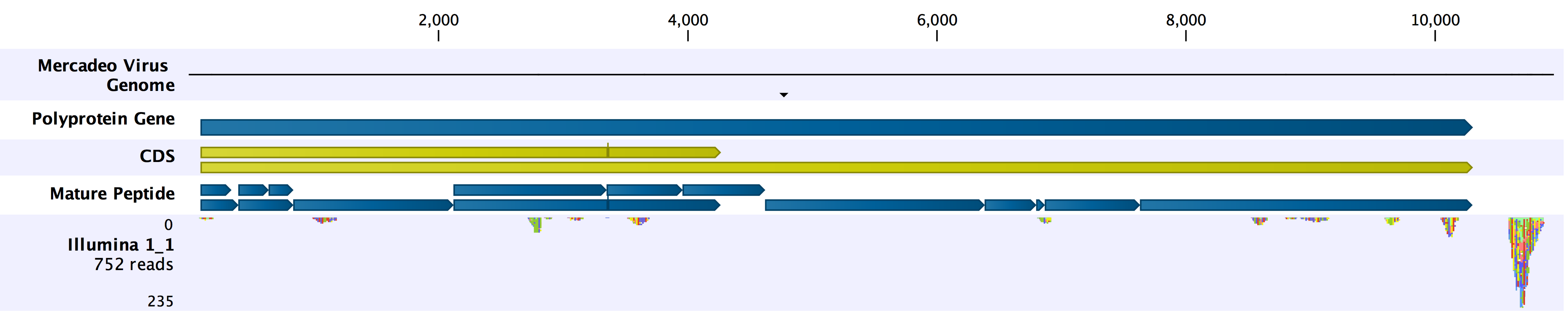


**Supplementary Figure S3:** Read mapping of the Illumina data from Sample 1.1 was carried out against 144 VEEV reference genomes (Supplemental Table 1 below), three Mercadeo Virus genomes (NC_027819, KP688058, KP688057), Choristoneura occidentalis granulovirus genome (NC_008168), and the Culex quinquefaciatus draft genome assembly (NZ_AAWU01000000). Zero reads mapped to any VEEV genome, but 752 reads mapped across the three MECDV genomes. The figure above shows these reads collectively remapped to a single MECDV reference (NC_027819). The same analysis of Illumina reads from Sample 4.1 showed zero reads aligning to MECDV, but 5,269 aligning across multiple VEEV genomes (see Figure 3 and Table 1 in the main text of the manuscript).

**Supplemental Table 1**

| Accession | Strain |
| --- | --- |
| AF075251 | EVEV Fe3-7c |
| AF075252 | Mena II |
| AF075257 | 78V-3531 |
| KC344432 | BT-2607 |
| KC344445 | 64U87 |
| KC344460 | 00SMM480-11 |
| KC344462 | 00SMM495-12 |
| KC344478 | MX09-M51 |
| KC344479 | VEEV MX09-M50 |
| KC344480 | MX09-M64 |
| KC344487 | VEEV 334250 |
| KC344493 | MX10-91M1 |
| KC344503 | 903104 |
| KC344516 | VEEV Beck-Wycoff |
| KC344519 | VEEV 307537 |
| KC344521 | 993MM304-1 |
| KC344525 | 75D143 |
| KF985959 | 1-Dec |
| KR260737 | EVG3-95 |
| L01442 | VEEV TrD |
| L04653 L00931 | P676 |

**Supplementary Table 2**

Sequencing reads classified at the family level by *kraken* are shown below for each of the sameples, and according to sequencing platform used.

| **ID** | **Illumina 1.1** | **Illumina 4.1** | **Nanopore 1.1** | **Nanopore 4.1** |
| --- | --- | --- | --- | --- |
| Acetobacteraceae | 197 | 2166 | 0 | 7 |
| Acholeplasmataceae | 8 | 7 | 1 | 1 |
| Acidimicrobiaceae | 18 | 9 | 0 | 0 |
| Acidithiobacillaceae | 18 | 283 | 0 | 2 |
| Acidobacteriaceae | 55 | 132 | 8 | 22 |
| Acidothermaceae | 1 | 1 | 0 | 0 |
| Actinomycetaceae | 6 | 0 | 1 | 0 |
| Adenoviridae | 1 | 0 | 0 | 0 |
| Aerococcaceae | 1 | 6 | 0 | 0 |
| Aeromonadaceae | 6 | 9 | 0 | 0 |
| Alcaligenaceae | 60 | 18 | 0 | 2 |
| Alcanivoracaceae | 102 | 14 | 0 | 0 |
| Alicyclobacillaceae | 1 | 0 | 0 | 0 |
| Alteromonadaceae | 51 | 2076 | 0 | 2 |
| Amoebophilaceae | 3 | 1 | 0 | 0 |
| Anaerolineaceae | 5 | 7 | 0 | 0 |
| Anaeromyxobacteraceae | 4 | 6 | 0 | 0 |
| Anaplasmataceae | 207 | 99 | 9 | 2 |
| Aquificaceae | 4 | 0 | 0 | 0 |
| Ascoviridae | 3 | 6 | 1 | 1 |
| Bacillaceae | 59 | 148 | 23 | 44 |
| Bacteriovoracaceae | 148 | 8 | 0 | 1 |
| Bacteroidaceae | 1 | 27 | 1 | 2 |
| Baculoviridae | 27862 | 98625 | 502 | 173 |
| Bartonellaceae | 3 | 4 | 0 | 0 |
| Bdellovibrionaceae | 36 | 10 | 0 | 0 |
| Beijerinckiaceae | 16 | 2 | 0 | 1 |
| Beutenbergiaceae | 29 | 19 | 1 | 0 |
| Bifidobacteriaceae | 65 | 65 | 1 | 1 |
| Blattabacteriaceae | 5 | 4 | 0 | 0 |
| Brachyspiraceae | 38 | 42 | 3 | 9 |
| Bradyrhizobiaceae | 134 | 1143 | 1 | 0 |
| Brucellaceae | 9 | 7 | 0 | 0 |
| Burkholderiaceae | 83 | 240 | 2 | 5 |
| Caldilineaceae | 1134 | 217 | 22 | 3 |
| Caldisericaceae | 1 | 7 | 0 | 0 |
| Caldisphaeraceae | 0 | 0 | 0 | 1 |
| Campylobacteraceae | 37 | 7 | 2 | 2 |
| Candidatus Midichloriaceae | 1 | 8 | 0 | 0 |
| Cardiobacteriaceae | 41 | 6 | 0 | 0 |
| Carnobacteriaceae | 1 | 10 | 0 | 0 |
| Catenulisporaceae | 1 | 0 | 0 | 0 |
| Caulobacteraceae | 4721 | 11 | 8 | 7 |
| Cellulomonadaceae | 1 | 1 | 0 | 0 |
| Chitinophagaceae | 68 | 3 | 0 | 7 |
| Chlamydiaceae | 5 | 2 | 0 | 1 |
| Chlorobiaceae | 97 | 8 | 1 | 0 |
| Chloroflexaceae | 0 | 1 | 0 | 0 |
| Chromatiaceae | 22 | 502 | 0 | 2 |
| Chromobacteriaceae | 10 | 17 | 0 | 0 |
| Chrysiogenaceae | 4 | 0 | 0 | 0 |
| Chthonomonadaceae | 2 | 7 | 1 | 0 |
| Clostridiaceae | 97 | 47 | 4 | 8 |
| Clostridiales Family XVII Incertae Sedis | 3 | 14 | 0 | 0 |
| Comamonadaceae | 2963 | 247 | 1 | 5 |
| Conexibacteraceae | 14 | 2 | 0 | 0 |
| Coriobacteriaceae | 16 | 27 | 2 | 1 |
| Corynebacteriaceae | 1684 | 738 | 9 | 7 |
| Coxiellaceae | 1 | 112 | 0 | 0 |
| Cryomorphaceae | 5 | 8 | 0 | 0 |
| Cyclobacteriaceae | 45 | 1 | 0 | 0 |
| Cystobacteraceae | 4 | 2 | 0 | 0 |
| Cytophagaceae | 1829 | 455 | 6 | 2 |
| Deferribacteraceae | 1 | 2 | 0 | 0 |
| Dehalococcoidaceae | 28 | 1 | 0 | 0 |
| Deinococcaceae | 8 | 15 | 0 | 0 |
| Dermabacteraceae | 184 | 5 | 1 | 0 |
| Dermacoccaceae | 237 | 0 | 1 | 0 |
| Desulfarculaceae | 1 | 1 | 0 | 0 |
| Desulfobacteraceae | 27 | 90 | 1 | 0 |
| Desulfobulbaceae | 17 | 13 | 1 | 1 |
| Desulfohalobiaceae | 1 | 1 | 0 | 0 |
| Desulfomicrobiaceae | 1 | 119 | 0 | 1 |
| Desulfovibrionaceae | 41 | 190 | 3 | 1 |
| Desulfurobacteriaceae | 4 | 3 | 0 | 0 |
| Desulfurococcaceae | 1 | 1 | 0 | 0 |
| Dictyoglomaceae | 1 | 2 | 0 | 0 |
| Ectothiorhodospiraceae | 30 | 231 | 0 | 0 |
| Elusimicrobiaceae | 3 | 2 | 0 | 0 |
| Enterobacteriaceae | 2065 | 3376 | 27 | 74 |
| Enterococcaceae | 0 | 14 | 0 | 0 |
| Entomoplasmataceae | 8 | 1 | 0 | 0 |
| Erysipelotrichaceae | 5 | 0 | 0 | 0 |
| Erythrobacteraceae | 14 | 0 | 0 | 0 |
| Eubacteriaceae | 5 | 12 | 0 | 0 |
| Fibrobacteraceae | 0 | 28 | 0 | 0 |
| Flammeovirgaceae | 0 | 1 | 0 | 0 |
| Flavobacteriaceae | 336 | 200 | 3 | 6 |
| Francisellaceae | 7 | 70 | 0 | 0 |
| Frankiaceae | 58 | 6 | 0 | 0 |
| Fusobacteriaceae | 11 | 27 | 0 | 0 |
| Gallionellaceae | 0 | 2 | 0 | 1 |
| Gemmatimonadaceae | 1 | 12 | 0 | 0 |
| Geobacteraceae | 15 | 47 | 3 | 9 |
| Geodermatophilaceae | 1 | 7 | 0 | 3 |
| Glycomycetaceae | 3 | 9 | 1 | 0 |
| Gordoniaceae | 25 | 0 | 0 | 0 |
| Hahellaceae | 0 | 1 | 0 | 0 |
| Halanaerobiaceae | 1 | 2 | 0 | 0 |
| Halobacteriaceae | 1 | 57 | 9 | 12 |
| Halobacteroidaceae | 6 | 2 | 0 | 1 |
| Halomonadaceae | 7 | 1399 | 5 | 18 |
| Halothiobacillaceae | 1 | 240 | 0 | 0 |
| Helicobacteraceae | 2019 | 17 | 49 | 25 |
| Heliobacteriaceae | 1 | 3 | 0 | 0 |
| Herpesviridae | 1 | 8 | 0 | 1 |
| Herpetosiphonaceae | 2 | 4 | 0 | 0 |
| Hydrogenophilaceae | 0 | 1 | 0 | 0 |
| Hydrogenothermaceae | 3 | 2 | 0 | 0 |
| Hyphomicrobiaceae | 49 | 7 | 0 | 1 |
| Hyphomonadaceae | 91 | 37 | 0 | 1 |
| Idiomarinaceae | 0 | 3 | 0 | 0 |
| Ignavibacteriaceae | 0 | 2 | 0 | 0 |
| Inoviridae | 0 | 1 | 0 | 0 |
| Intrasporangiaceae | 48 | 0 | 0 | 0 |
| Jonesiaceae | 8 | 9 | 0 | 1 |
| Kineosporiaceae | 4 | 1 | 1 | 0 |
| Kofleriaceae | 6 | 34 | 0 | 3 |
| Lachnospiraceae | 12 | 0 | 0 | 0 |
| Lactobacillaceae | 29 | 69 | 9 | 14 |
| Legionellaceae | 11 | 1328 | 0 | 3 |
| Leptospiraceae | 30 | 51 | 0 | 0 |
| Leptotrichiaceae | 12 | 4 | 0 | 0 |
| Leuconostocaceae | 4 | 6 | 0 | 0 |
| Leviviridae | 3 | 0 | 0 | 0 |
| Listeriaceae | 2 | 2 | 0 | 1 |
| Melioribacteraceae | 0 | 8 | 0 | 0 |
| Methanocaldococcaceae | 0 | 0 | 0 | 3 |
| Methanocellaceae | 4 | 3 | 0 | 0 |
| Methanococcaceae | 0 | 2 | 5 | 4 |
| Methanomicrobiaceae | 0 | 3 | 0 | 0 |
| Methanoregulaceae | 0 | 2 | 0 | 0 |
| Methanosarcinaceae | 6 | 10 | 2 | 4 |
| Methanothermaceae | 0 | 0 | 1 | 1 |
| Methylacidiphilaceae | 2 | 113 | 0 | 0 |
| Methylobacteriaceae | 165 | 44 | 0 | 2 |
| Methylococcaceae | 14 | 246 | 0 | 1 |
| Methylocystaceae | 14 | 2 | 0 | 0 |
| Methylophilaceae | 4 | 11 | 0 | 0 |
| Microbacteriaceae | 64 | 80 | 0 | 3 |
| Micrococcaceae | 1247 | 297 | 11 | 6 |
| Micromonosporaceae | 16 | 22 | 0 | 0 |
| Microviridae | 1 | 0 | 0 | 0 |
| Moraxellaceae | 630 | 91 | 2 | 2 |
| Mycobacteriaceae | 121 | 23 | 0 | 1 |
| Mycoplasmataceae | 105 | 54 | 1 | 4 |
| Myoviridae | 0 | 0 | 1 | 0 |
| Myxococcaceae | 340 | 12 | 1 | 0 |
| Nakamurellaceae | 3 | 0 | 0 | 0 |
| Natranaerobiaceae | 0 | 3 | 0 | 0 |
| Nautiliaceae | 0 | 1 | 0 | 0 |
| Neisseriaceae | 83 | 94 | 2 | 1 |
| Nimaviridae | 1 | 0 | 0 | 0 |
| Nitrosomonadaceae | 20 | 13 | 0 | 0 |
| Nitrosopumilaceae | 0 | 2 | 0 | 0 |
| Nitrososphaeraceae | 1 | 1 | 0 | 0 |
| Nitrospiraceae | 8 | 11 | 0 | 0 |
| Nocardiaceae | 33 | 16 | 1 | 0 |
| Nocardioidaceae | 47 | 8 | 0 | 0 |
| Nocardiopsaceae | 30 | 14 | 0 | 0 |
| Nostocaceae | 52 | 79 | 0 | 6 |
| Nudiviridae | 0 | 2 | 0 | 0 |
| Oceanospirillaceae | 2 | 821 | 0 | 4 |
| Opitutaceae | 0 | 33 | 0 | 5 |
| Oscillospiraceae | 2 | 0 | 0 | 0 |
| Oxalobacteraceae | 9 | 73 | 0 | 2 |
| Paenibacillaceae | 13 | 8 | 0 | 0 |
| Parachlamydiaceae | 21 | 13 | 0 | 0 |
| Parvularculaceae | 1 | 0 | 0 | 0 |
| Pasteurellaceae | 8 | 1199 | 3 | 12 |
| Pelobacteraceae | 0 | 14 | 0 | 0 |
| Peptococcaceae | 1561 | 407 | 9 | 7 |
| Peptoniphilaceae | 1 | 6 | 0 | 0 |
| Peptostreptococcaceae | 1 | 29 | 9 | 20 |
| Phycisphaeraceae | 1 | 20 | 0 | 0 |
| Phycodnaviridae | 0 | 0 | 12 | 38 |
| Phyllobacteriaceae | 39 | 27 | 1 | 5 |
| Picrophilaceae | 0 | 1 | 0 | 1 |
| Piscirickettsiaceae | 5 | 27 | 0 | 1 |
| Planctomycetaceae | 117 | 169 | 2 | 0 |
| Planococcaceae | 0 | 0 | 0 | 1 |
| Polyangiaceae | 9 | 119 | 0 | 5 |
| Polydnaviridae | 0 | 1 | 1 | 2 |
| Porphyromonadaceae | 54 | 29 | 0 | 0 |
| Potyviridae | 0 | 0 | 0 | 1 |
| Poxviridae | 1 | 13 | 9 | 28 |
| Prevotellaceae | 3 | 64 | 0 | 1 |
| Prochlorococcaceae | 3 | 1 | 0 | 0 |
| Promicromonosporaceae | 4 | 0 | 0 | 0 |
| Propionibacteriaceae | 371 | 1566 | 6 | 14 |
| Pseudoalteromonadaceae | 3 | 1 | 1 | 0 |
| Pseudomonadaceae | 124 | 192 | 7 | 10 |
| Pseudonocardiaceae | 83 | 8 | 0 | 0 |
| Psychromonadaceae | 6 | 1 | 0 | 0 |
| Puniceicoccaceae | 8 | 1 | 0 | 0 |
| Retroviridae | 1 | 4 | 1 | 0 |
| Rhizobiaceae | 218 | 57 | 1 | 4 |
| Rhodobacteraceae | 545 | 31 | 2 | 2 |
| Rhodobiaceae | 7 | 5 | 0 | 0 |
| Rhodocyclaceae | 21 | 18 | 0 | 0 |
| Rhodospirillaceae | 89 | 54 | 2 | 2 |
| Rhodothermaceae | 4 | 2 | 0 | 0 |
| Rickettsiaceae | 12 | 11 | 1 | 0 |
| Rikenellaceae | 0 | 6 | 0 | 0 |
| Rivulariaceae | 30 | 0 | 0 | 0 |
| Roseiflexaceae | 1 | 1 | 0 | 1 |
| Rubrobacteraceae | 12 | 0 | 0 | 1 |
| Ruminococcaceae | 15 | 575 | 13 | 25 |
| Sanguibacteraceae | 43 | 1 | 0 | 1 |
| Saprospiraceae | 11 | 22 | 0 | 2 |
| Segniliparaceae | 41 | 40 | 0 | 0 |
| Shewanellaceae | 1 | 10 | 1 | 2 |
| Simkaniaceae | 11 | 3 | 0 | 0 |
| Siphoviridae | 2 | 25 | 0 | 0 |
| Solibacteraceae | 6 | 22 | 0 | 1 |
| Sphaerobacteraceae | 0 | 3 | 0 | 0 |
| Sphingobacteriaceae | 19 | 14 | 0 | 0 |
| Sphingomonadaceae | 179 | 21 | 1 | 18 |
| Spirochaetaceae | 552 | 915 | 11 | 2 |
| Spiroplasmataceae | 1261 | 2 | 21 | 0 |
| Sporolactobacillaceae | 0 | 2 | 0 | 0 |
| Staphylococcaceae | 412 | 386 | 0 | 4 |
| Streptococcaceae | 309 | 2053 | 1 | 7 |
| Streptomycetaceae | 61 | 36 | 0 | 2 |
| Streptosporangiaceae | 3 | 6 | 5 | 4 |
| Sulfolobaceae | 4 | 12 | 0 | 0 |
| Sulfuricellaceae | 3 | 14 | 0 | 0 |
| Symbiobacteriaceae | 26 | 0 | 0 | 0 |
| Synergistaceae | 1385 | 334 | 15 | 5 |
| Syntrophaceae | 4 | 5 | 0 | 1 |
| Syntrophobacteraceae | 0 | 9 | 0 | 0 |
| Syntrophomonadaceae | 0 | 0 | 0 | 1 |
| Thermaceae | 32 | 4 | 1 | 0 |
| Thermoanaerobacteraceae | 5 | 5 | 0 | 0 |
| Thermoanaerobacterales Family III Incertae Sedis | 15 | 1 | 2 | 3 |
| Thermoanaerobacterales Family IV Incertae Sedis | 19 | 1 | 0 | 0 |
| Thermodesulfobiaceae | 13 | 5 | 0 | 0 |
| Thermomicrobiaceae | 15 | 47 | 0 | 0 |
| Thermomonosporaceae | 2 | 6 | 0 | 0 |
| Thermoplasmataceae | 0 | 0 | 4 | 0 |
| Thermoproteaceae | 2 | 5 | 3 | 0 |
| Thermotogaceae | 43 | 30 | 0 | 0 |
| **Togaviridae** | **0** | **796** | **0** | **1** |
| Trueperaceae | 0 | 16 | 0 | 0 |
| Tsukamurellaceae | 0 | 8 | 0 | 0 |
| Veillonellaceae | 62 | 61 | 0 | 1 |
| Verrucomicrobiaceae | 3 | 1 | 0 | 0 |
| Vibrionaceae | 22 | 459 | 4 | 9 |
| Xanthobacteraceae | 34 | 17 | 0 | 0 |
| Xanthomonadaceae | 1569 | 544 | 4 | 2 |

**List of Suggested Hardware and Consumables for Field-Forward Agnostic Nanopore Sequencing for the Purposes of Environmental Biosurveillance --------**

**1)** *the Biomeme Bulk Nucleic Acid Extraction kit provides individually wrapped packets containing all plastic-ware needed for each sample (syringe assembly, tubing, plastic pestle, 2 ml elution tube) and 4 reagent bottles (2 x 15 ml, 2 x 30 ml)*

**2)** *the Biomeme two3 thermocycler (or other portable thermocycler: e.g., MIC, miniPCR, etc.) and accompanying 0.2 ml RT-qPCR tubes with lyophilized assays*

**3)** *the MinION nanopore sequencing device*

**4)** *required number of R9.4 flowcells*

**5)** *ONT sequencing library preparation kit (SQK-LSK108) and flow cell wash kit (EXP-WSH002), and associated reagents*

**6)** *GeneRead rRNA Depletion Kit*

**7)** *Sigma WTA2 Whole Transcriptome Amplification Kit*

**8)** *50 ml conical tube filled with 70% EtOH (1). 50 ml conical tube filled with 100% EtOH (1). 50 ml conical tube of molecular grade H_2_0 (1).*

**9)** *mini centrifuge*

**10)** *P1000, P200, P20, P10 pipetman and tip boxes*

**11)** *magnetic tube stand*

**12)** *AMPure and Streptavidin beads (2 x 5 ml bottles)*

**13)** *Intel NUC Skull Canyon (32GB RAM, up to 2TB SSD, hyper-threaded quad-core, Ubuntu 16.04 LTS) and Bluetooth monitor/keyboard/trackpad*

**List of VEEV Reference Genome Accession Numbers from Targeted Read-Mapping Database --------**

| AF004458 | AY741139 | KC344441 | KC344461 | KC344481 | KC344501 | KC344521 | VEU55347 |
| --- | --- | --- | --- | --- | --- | --- | --- |
| AF004459 | AY823299 | KC344442 | KC344462 | KC344482 | KC344502 | KC344522 | VEU55350 |
| AF004472 | DQ390224 | KC344443 | KC344463 | KC344483 | KC344503 | KC344523 | VEU55360 |
| AF069903 | EEVCOMGEN | KC344444 | KC344464 | KC344484 | KC344504 | KC344524 | VEU55362 |
| AF075251 | EEVNSPECFA | KC344445 | KC344465 | KC344485 | KC344505 | KC344525 |  |
| AF075252 | EEVNSPENV | KC344446 | KC344466 | KC344486 | KC344506 | KC344526 |  |
| AF075253 | EEVNSPEPA | KC344447 | KC344467 | KC344487 | KC344507 | KC344527 |  |
| AF075254 | EEVNSPEPB | KC344448 | KC344468 | KC344488 | KC344508 | KC344528 |  |
| AF075255 | KC344429 | KC344449 | KC344469 | KC344489 | KC344509 | KC344529 |  |
| AF075256 | KC344430 | KC344450 | KC344470 | KC344490 | KC344510 | KC344530 |  |
| AF075257 | KC344431 | KC344451 | KC344471 | KC344491 | KC344511 | KC344531 |  |
| AF075258 | KC344432 | KC344452 | KC344472 | KC344492 | KC344512 | KF985959 |  |
| AF075259 | KC344433 | KC344453 | KC344473 | KC344493 | KC344513 | KJ410017 |  |
| AF100566 | KC344434 | KC344454 | KC344474 | KC344494 | KC344514 | KP282671 |  |
| AF375051 | KC344435 | KC344455 | KC344475 | KC344495 | KC344515 | KR260736 |  |
| AF448535 | KC344436 | KC344456 | KC344476 | KC344496 | KC344516 | KR260737 |  |
| AF448536 | KC344437 | KC344457 | KC344477 | KC344497 | KC344517 | NC_001449 | |
| AF448537 | KC344438 | KC344458 | KC344478 | KC344498 | KC344518 | VEU34999 |  |
| AF448538 | KC344439 | KC344459 | KC344479 | KC344499 | KC344519 | VEU55342 |  |
| AF448539 | KC344440 | KC344460 | KC344480 | KC344500 | KC344520 | VEU55345 |  |
